# Supplementary material for: Meal scheduling corrects obesogenic diet induced-uncoupling of cortico-hippocampal activities supporting memory
Source: eBioMedicine. 2025 Jun 16;117:105783. doi: 10.1016/j.ebiom.2025.105783 (PMC12209983; doi:10.1016/j.ebiom.2025.105783)
Supplement: Supplementary Figures [file mmc2.docx]

**Study design**

The protocol was not pre-registered.

Animals were randomly allocated to NC or HF ad lib groups.

No effect of sex was noted. So we pooled males and females, further distributed randomly to the following groups:

- NC TRF
- NC ad lib
- HF ad lib
- HF TRF

The outcome measures are as follows:

- Feeding rhythm
- Calorie intake
- object recognition memory
- freezing behaviour
- locomotor activity
- anxiety in the open field
- body weight
- dendritic spine addition
- elimination
- survival
- Cfos+ cell number
- Cfos+YFP+ cell number
- Cfos+Tomato+ cell number
- Tomato cell number
- pS134 levels
- pS226 levels
- GR levels.

Rationale for repeated measures as follows:

- Longitudinal studies to track impairment with HF and reversal with TRF.
- Chemogenetic to determine functional interaction of transgene and CNO. Multisite combinations to assess dominant effect between S1 and CA1.

The numbers of repeated measures is as follow:

- NOR behaviour was performed 3 times at week 1, 8, 12 post-weaning.
- Fear conditioning was performed once at week 13 post-weaning.
- Neuroimaging was performed 4 times in cortex at week 1, 8, 12, 13 post-weaning.
- Neuroimaging was performed 3 times in CA1 at week 8, 12, 13 post-weaning.
- RU486/vehicle injection was performed once at week 12 post-weaning.
- CNO/vehicle injection was performed once at week 12 post-weaning.

There are 13 experiments with the following group repartition:

- Experiment 1 has 4 groups: NC ad lib, NC TRF, HF ad lib, HF TRF to test object memory 3 times and emotional memory once.
- Experiment 2 has 4 groups: NC ad lib, NC TRF, HF ad lib, HF TRF for neuroimaging 4 times.
- Experiment 3 has 4 groups: NC ad lib, NC TRF, HF ad lib, HF TRF for post-mortem histology in thy1YFP.
- Experiment 4 has 4 groups: NC ad lib, NC TRF, HF ad lib, HF TRF for post-mortem histology in FosTRAP2;Ai14 mice for engram capture.
- Experiment 5 has 4 groups: NC ad lib +vehicle, NC ad lib +CNO, HF ad lib + vehicle, HF ad lib +CNO for DREADD-Gq in S1 on object memory.
- Experiment 6 has 4 groups: NC ad lib +vehicle, NC ad lib +CNO, HF ad lib + vehicle, HF ad lib +CNO for DREADD-Gi in CA1 on object memory.
- Experiment 7 has 6 groups: HF ad lib sham +vehicle, HF ad lib sham +CNO, HF ad lib Gi+vehicle, HF ad lib Gi+CNO, HF ad lib Gq+ vehicle, HF ad lib Gq+CNO for object memory.
- Experiment 8 has 4 groups: NC ad lib+vehicle, NC ad lib+RU486, HF ad lib+vehicle, HF ad lib+RU486 to test object memory.
- Experiment 9 has 4 groups: NC ad lib+vehicle, NC ad lib+RU486 imaged in S1 and NC ad lib+vehicle, NC ad lib+RU486 imaged in CA1.
- Experiment 10 has 4 groups: NC ad lib, NC TRF, HF ad lib, HF TRF for histology in S1 and CA1.
- Experiment 11 has 8 groups: WT NC ad lib, WT NC TRF, WT HF ad lib, WT HF TRF and mutant NC ad lib, mutant NC TRF, mutant HF ad lib, mutant HF TRF for histology in S1 and CA1.
- Experiment 12 has 8 groups: WT NC ad lib, WT NC TRF, WT HF ad lib, WT HF TRF and mutant NC ad lib, mutant NC TRF, mutant HF ad lib, mutant HF TRF for object memory.
- Experiment 13 has 8 groups: WT NC ad lib, WT NC TRF, WT HF ad lib, WT HF TRF and mutant NC ad lib, mutant NC TRF, mutant HF ad lib, mutant HF TRF for emotional memory.

**Supplementary Figures**


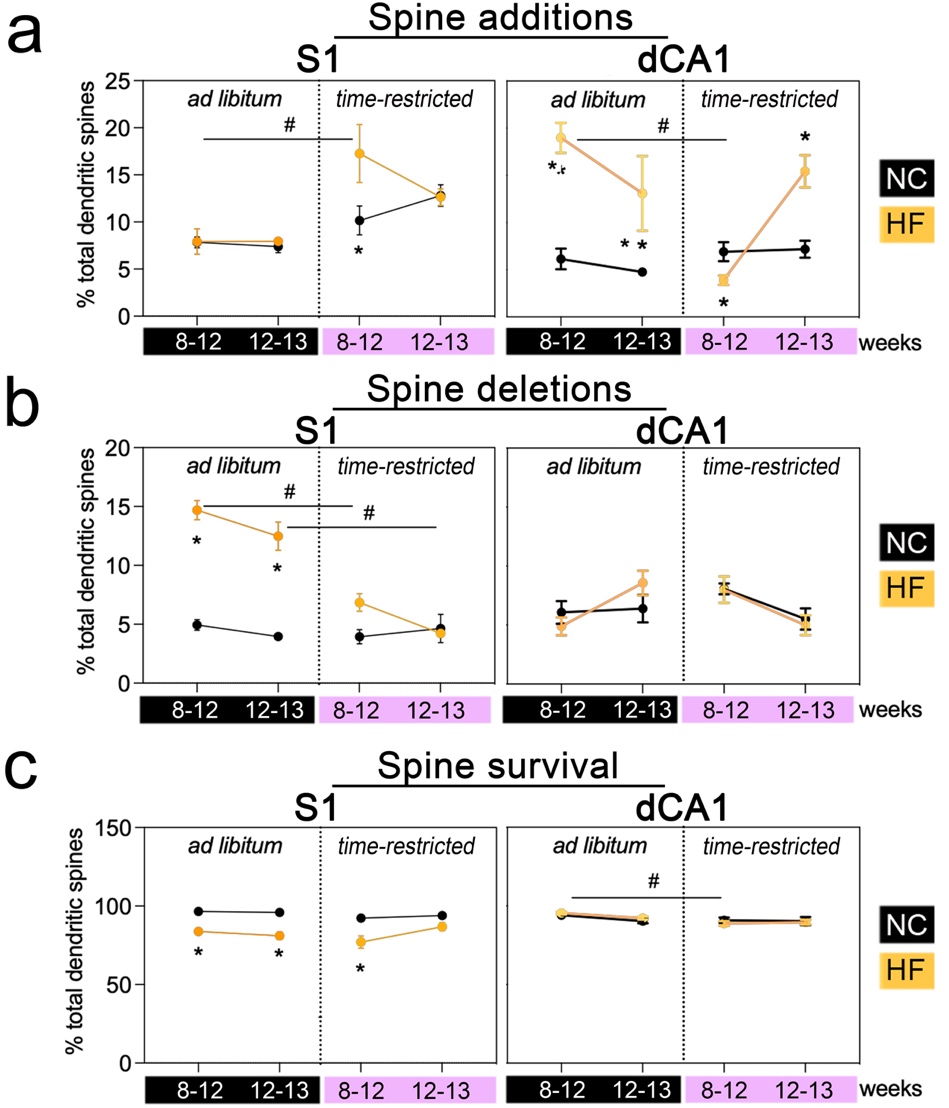


**Supplementary Figure 1. TRF resets bi-directional spine remodeling in S1 and dCA1 of mice fed HFS food.**

**a.** % Spine additions in somatosensory cortex S1 and dorsal hippocampus subfield dCA1 between week 8 and 12, and between week 12 and 13 acquired in vivo by 2-photon microcopy in *Thy1*-YFP males only. Data are means ± SEM of n = 6 mice/group. Data has normal distribution (Shapiro-Wilk test *p* > 0.05) and homoscedasticity (Levene’s test *p* > 0.05). Three-way ANOVA for *S1*: Effect of time *F*_(1,20)_ = 0.2 *p* = 0.6, schedule *F*_(1,20)_ = 23 *p* < 0.0001, diet *F*_(1,20)_ = 3.1 *p* = 0.09, time x schedule *F*_(1,20)_ = 0.06 *p* = 0.8, time x diet *F*_(1,20)_ = 3.4 *p* = 0.08, schedule x diet *F*_(1,20)_ = 3.2 *p* = 0.08, time x schedule x diet *F*_(1,20)_ = 4.5 *p* = 0.04. Post-hoc comparisons with Sidak test: NC restricted vs HFS restricted ^*^*p* = 0.01; HFS ad lib vs HFS restricted ^#^*p* = 0.005. For *CA1*: Effect of time *F*_(1,20)_ = 1.7 *p* = 0.2, schedule *F*_(1,20)_ = 5.4 *p* = 0.03, diet *F*_(1,20)_ = 46 *p* < 0.0001, time x schedule *F*_(1,20)_ = 28 *p* < 0.0001, time x diet *F*_(1,20)_ = 2 *p* = 0.1, schedule x diet *F*_(1,20)_ = 16 *p* = 0.0007, time x schedule x diet *F*_(1,20)_ = 17 *p* = 0.0004. Post-hoc comparisons with Sidak test: NC ad lib vs HFS ad lib ^*^*p* < 0.0001 and ^*^*p* = 0.0005; NC restricted vs HFS restricted ^*^*p* < 0.0001; HFS ad lib vs HFS restricted ^#^*p* = 0.02 and ^#^*p* < 0.0001. HF: high fat/sugar diet, NC: normal chow. Black color: ad libitum, pink color: time restricted feeding.

**b.** % Spine eliminations in somatosensory cortex S1 and dorsal hippocampus subfield dCA1 between week 8 and 12, and between week 12 and 13 acquired in vivo by 2-photon microcopy in *Thy1*-YFP males only. Data are means ± SEM of n = 6 mice/group. Data has normal distribution (Shapiro-Wilk test *p* > 0.05) and homoscedasticity (Levene’s test *p* > 0.05). Three-way ANOVA mixed-effect *for S1*: Effect of time *F*_(1,40)_ = 8 *p* = 0.005, schedule *F*_(1,40)_ = 83 *p* < 0.0001, diet *F*_(1,22)_ = 143 *p* < 0.0001, time x schedule *F*_(1,40)_ = 1.9 *p* = 0.1, time x diet *F*_(1,40)_ = 5.3 *p* = 0.02, schedule x diet *F*_(1,40)_ = 91 *p* < 0.0001, time x schedule x diet *F*_(1,40)_ = 1.6 *p* = 0.2. Post-hoc comparisons with Sidak test: NC ad lib vs HFS ad lib ^*^*p* < 0.0001; HFS ad lib vs HFS restricted ^#^*p* < 0.0001. For *CA1*: Effect of time *F*_(1,20)_ = 1.4 *p* = 0.2, schedule *F*_(1,20)_ = 0.5 *p* = 0.4, diet *F*_(1,20)_ = 0.3 *p* = 0.5, time x schedule *F*_(1,20)_ = 9.4 *p* = 0.006, time x diet *F*_(1,20)_ = 0.3 *p* = 0.5, schedule x diet *F*_(1,20)_ = 0.12 *p* = 0.7, time x schedule x diet *F*_(1,20)_ = 3.4 *p* = 0.07. No difference in post-hoc comparisons with Sidak test. HF: high fat/sugar diet, NC: normal chow. Black color: ad libitum, pink color: time restricted feeding.

**c.** % Spine survival in somatosensory cortex S1 and dorsal hippocampus subfield dCA1 between week 8 and 12, and between week 12 and 13 acquired in vivo by 2-photon microcopy in *Thy1*-YFP males only. Data are means ± SEM of n = 6 mice/group. Data has normal distribution (Shapiro-Wilk test *p* > 0.05) and homoscedasticity (Levene’s test *p* > 0.05). Three-way ANOVA *for S1*: Effect of time *F*_(1,40)_ = 4.9 *p* = 0.03, schedule *F*_(1,40)_ = 1.7 *p* = 0.2, diet *F*_(1,40)_ = 73 *p* < 0.0001, time x schedule *F*_(1,40)_ = 6.9 *p* = 0.01, time x diet *F*_(1,40)_ = 2.5 *p* = 0.1, schedule x diet *F*_(1,40)_ = 1.5 *p* = 0.2, time x schedule x diet *F*_(1,40)_ = 4.1 *p* = 0.04. Post-hoc comparisons with Sidak test: NC ad lib vs HFS ad lib ^*^*p* < 0.001; HFS ad lib vs HFS restricted ^#^*p* = 0.001 and ^#^*p* = 0.04. For *CA1*: Effect of time *F*_(1,20)_ = 1.1 *p* = 0.2, schedule *F*_(1,20)_ = 9.8 *p* = 0.005, diet *F*_(1,20)_ = 0.02 *p* = 0.8, time x schedule *F*_(1,20)_ = 4.1 *p* = 0.055, time x diet *F*_(1,20)_ = 0.8 *p* = 0.3, schedule x diet *F*_(1,20)_ = 3.6 *p* = 0.07, time x schedule x diet *F*_(1,20)_ = 1.3 *p* = 0.2. Post-hoc comparisons with Sidak test: HFS ad lib vs HFS restricted ^#^*p* = 0.002. HF: high fat/sugar diet, NC: normal chow. Black color: ad libitum, pink color: time restricted feeding.


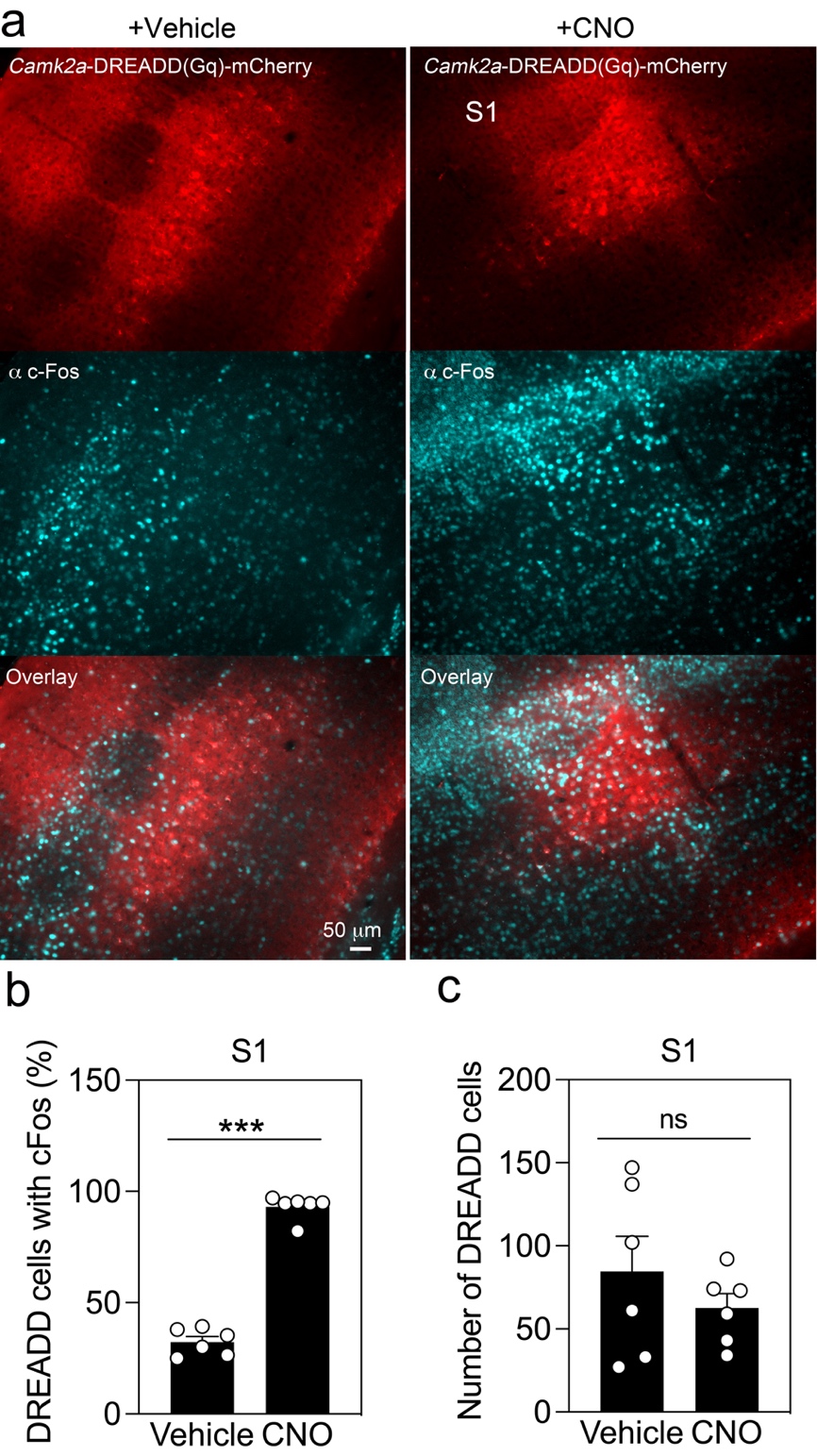


**Supplementary Figure 2. Chemogenetic activation of pyramidal neurons in S1.**

**a.** Viral-mediated expression of *Camk2a*-DREADD(Gq)-mCherry in the somatosensory cortex S1 and cFos induction 45 min after I.P. injection of Clozapine-N-oxide (CNO as DREADD ligand) or vehicle as control.

**b.** % DREADD(Gq)-positive cells co-labelled with cFos antibodies. Data are means ± SEM (n = 6 males injected I.P. with vehicle, 6 males injected I.P. with CNO). Data has normal distribution (Shapiro-Wilk test *p* > 0.05) and homoscedasticity (Spearman’s test *p* > 0.05). Statistical analysis done with 2-sided unpaired t-test ^***^*p* < 0.0001.

**c.** Number of DREADD(Gq)-positive cells. Data are means ± SEM (n = 6 males injected I.P. with vehicle, 6 males injected I.P. with CNO). Data has normal distribution (Shapiro-Wilk test *p* > 0.05) and homoscedasticity (Spearman’s test *p* > 0.05). Statistical analysis done with 2-sided unpaired t-test *p* = 0.36.


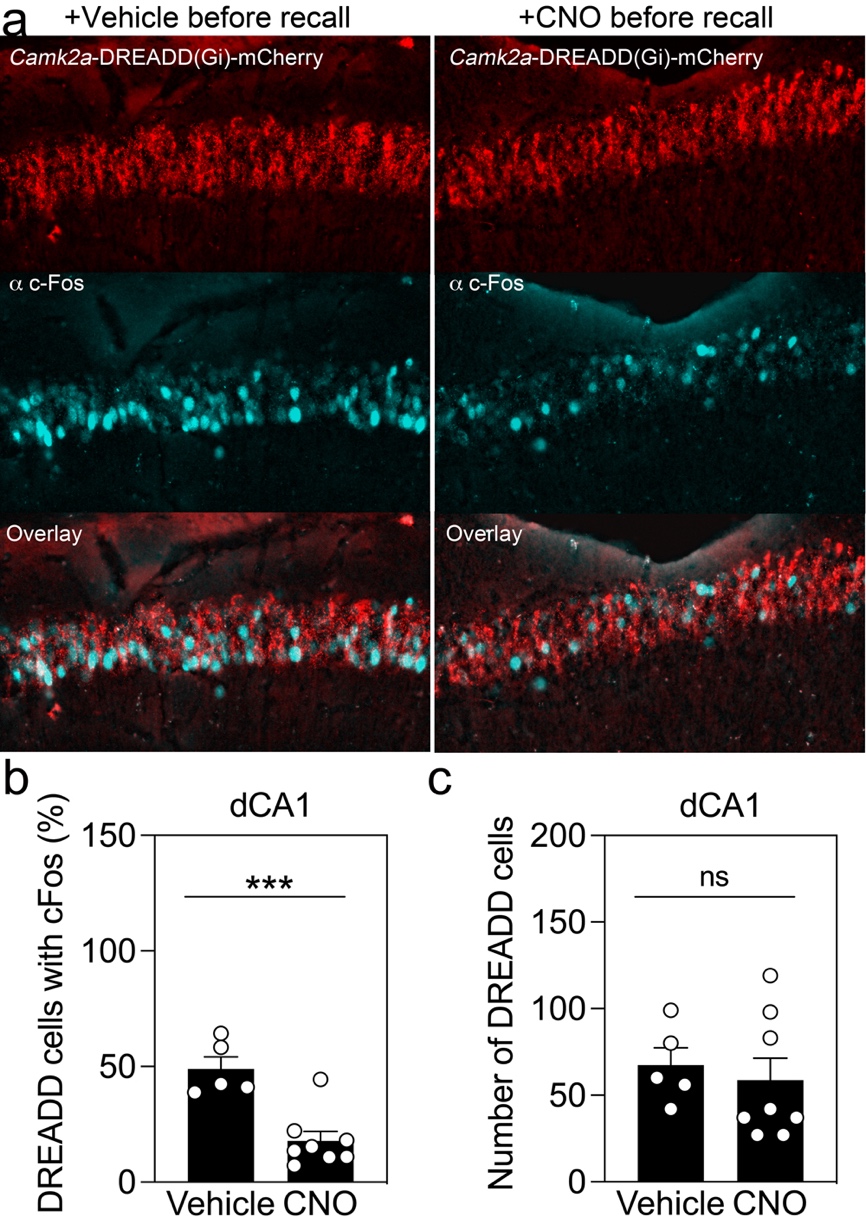


**Supplementary Figure 3. Chemogenetic inhibition of pyramidal neurons in dCA1.**

**a.** Viral-mediated expression of *Camk2a*-DREADD(Gi)-mCherry in dorsal hippocampus subfield dCA1 and cFos induction 1 hr after object memory recall and I.P. injection of Clozapine-N-oxide (CNO) or vehicle as control.

**b.** % DREADD(Gq)-positive cells co-labelled with cFos antibodies. Data are means ± SEM (n = 5 males injected I.P. with vehicle, 8 males injected I.P. with CNO). Data has normal distribution (Shapiro-Wilk test *p* > 0.05) and homoscedasticity (Spearman’s test *p* > 0.05). Statistical analysis done with 2-sided unpaired t-test ^***^*p* = 0.0007.

**c.** Number of DREADD(Gq)-positive cells. Data are means ± SEM (n = 5 males injected I.P. with vehicle, 8 males injected I.P. with CNO). Data has normal distribution (Shapiro-Wilk test *p* > 0.05) and homoscedasticity (Spearman’s test *p* > 0.05). Statistical analysis done with 2-sided unpaired t-test *p* = 0.64.

**
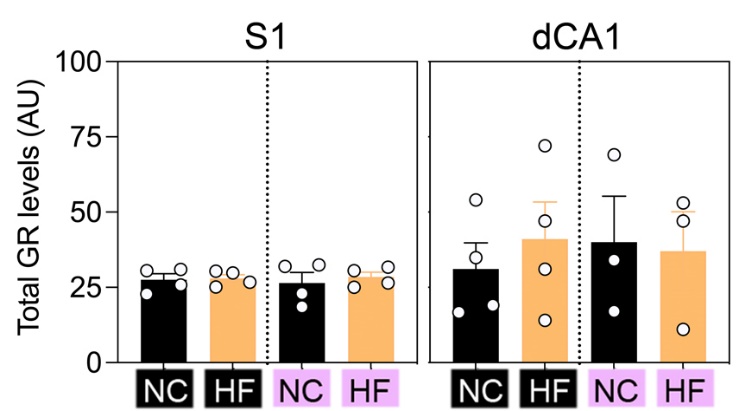
**

**Supplementary Figure 4. No effect of diet and meal scheduling on GR levels in S1 and dCA1**

Intensity (arbitrary unit as AU) of GR immunoreactivity in S1 (n = 16) and dCA1 (n = 14) in male mice fed NC or HFS-diet either *ad libitum* or on scheduling (means ± SEM). Data has normal distribution (Shapiro-Wilk test *p* > 0.05) and homoscedasticity (Spearman’s test *p* > 0.05). Three-way ANOVA: Effect of regions *F*_(1,22)_ = 2.8 *p* = 0.1, effect of regimen *F*_(1,22)_ = 0.03 *p* = 0.8, effect of diet *F*_(1,22)_ = 0.16 *p* = 0.6, effect of regimen x regions *F*_(1,22)_ = 0.05 *p* = 0.8, effect of regions x diet *F*_(1,22)_ = 0.03 *p* = 0.8, effect of regimen x diet *F*_(1,22)_ = 0.2 *p* = 0.6, effect of regimen x diet x regions *F*_(1,22)_ = 0.3 *p* = 0.5. HF: high fat/sugar diet, NC: normal chow. Black color: ad libitum, pink color: time restricted feeding.

**
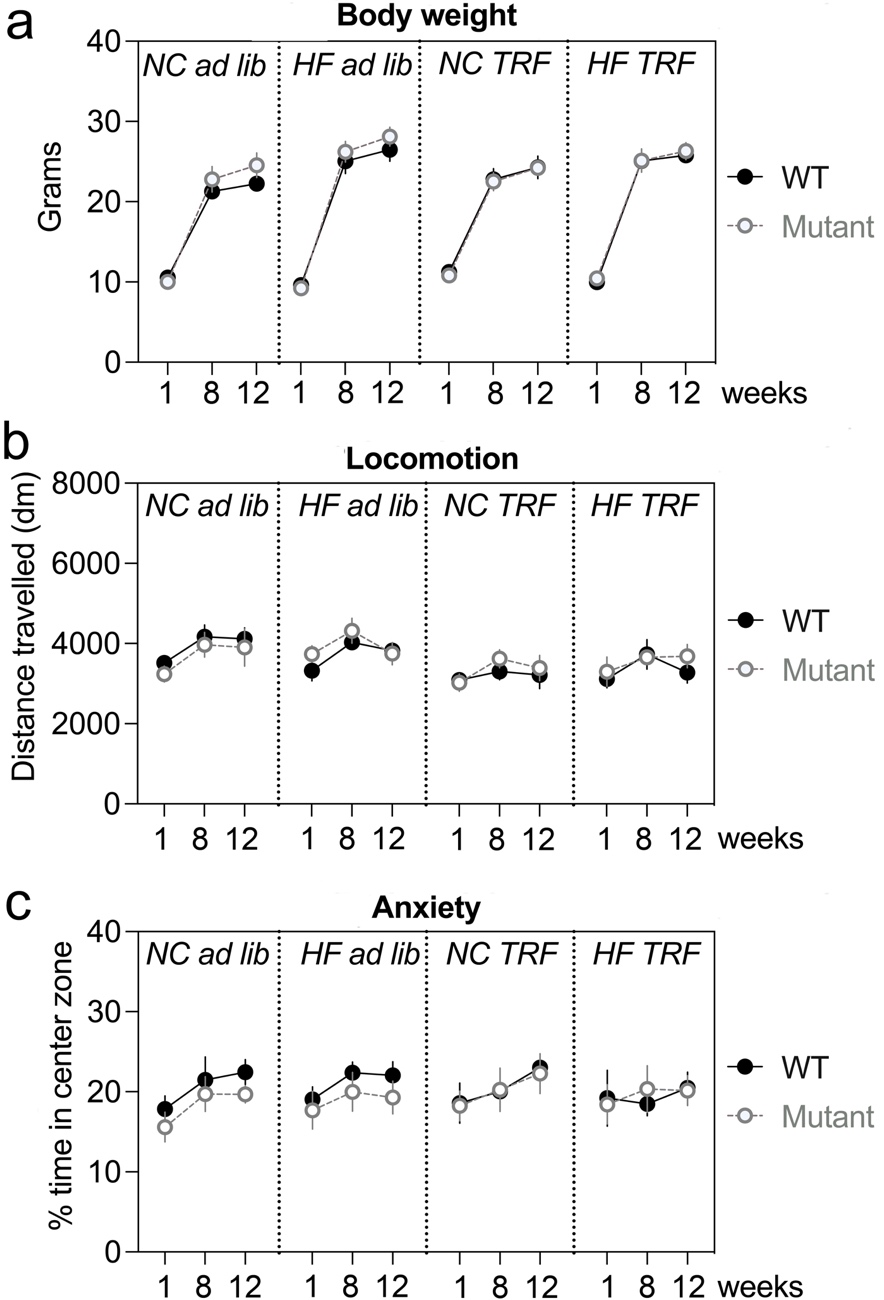
**

**Supplementary Figure 5. No effect of genotype (GR^S134A^) on locomotion, anxiety and body weight**

**a.** Developmental gain of body weight as a function of diet change (between week 1 and 8) and regimen change (between week 8 and 12). Data are means ± SEM. Repartition of 45 WT (GR^S134/S134^) homozygotes is as follows: n = 6 males + 6 females with NC ad libitum, 7 males + 7 females with HF ad libitum, 6 males + 6 females with NC restricted, 7 males + 4 females with HF restricted. Repartition of 43 mutants (GR^A134/A134^) homozygotes is as follows: n = 5 males + 5 females 5 KI males with NC ad libitum, 4 males + 6 females with HF ad libitum, 5 males + 4 females with NC restricted, 7 males + 6 females with HF restricted. Data has normal distribution (Shapiro-Wilk test *p* = 0.47). The use of the chi-square distribution instead of the F-distribution is justified by the lack of homoscedasticity (Levene’s test *p* < 0.0001). Five-way ANOVA (model type III): Chisq analysis: Effect of sex *χ2*_(1,68)_ = 1.8 *p* = 0.1, diet *χ2*_(1,68)_ = 0.9 *p* = 0.3, TRF *χ2*_(1,68)_ = 0.3 *p* = 0.5, time *χ2*_(1,68)_ = 241 *p* < 0.0001, genotype *χ2*_(1,68)_ = 1 *p* = 0.3, diet x sex *χ2*_(1,68)_ = 0.2 *p* = 0.5, diet x TRF *χ2*_(1,68)_ = 0.1 *p* = 0.68, sex x time *χ2*_(2,135)_ = 36 *p* < 0.0001, diet x time *χ2*_(2,135)_ = 2.8 *p* = 0.2, TRF x time *χ2*_(2,135)_ = 7.5 *p* = 0.002, sex x genotype *χ2*_(1,68)_ = 2.8 *p* = 0.09, diet x genotype *χ2*_(1,68)_ = 2.1 *p* = 0.1, TRF x genotype *χ2*_(1,68)_ = 0.02 *p* = 0.1, genotype x time *χ2*_(2,135)_ = 0.03 *p* = 0.9, sex x diet x TRF *χ2*_(1,68)_ = 0.9 *p* = 0.3, sex x diet x time *χ2*_(2,135)_ = 9 *p* = 0.01, sex x TRF x time *χ2*_(2,135)_ = 1.6 *p* = 0.4, diet x TRF x time *χ2*_(2,135)_ = 2.2 *p* = 0.3, sex x diet x genotype *χ2*_(1,68)_ = 4.5 *p* = 0.03, sex x TRF x genotype *χ2*_(1,68)_ = 0.1 *p* = 0.7, diet x genotype x TRF *χ2*_(1,68)_ = 0.03 *p* = 0.8, sex x genotype x time *χ2*_(2,135)_ = 7 *p* = 0.02, sex x diet x time *χ2*_(2,135)_ = 9 *p* = 0.01, sex x TRF x time *χ2*_(2,135)_ = 1.6 *p* = 0.4, diet x TRF x time *χ2*_(2,135)_ = 2.2 *p* = 0.3, sex x diet x genotype *χ2*_(1,68)_ = 4.5 *p* = 0.03, diet x time x genotype *χ2*_(2,135)_ = 1.3 *p* = 0.5, time x genotype x TRF *χ2*_(2,135)_ = 3.2 *p* = 0.1, sex x diet x TRF x time *χ2*_(2,135)_ = 0.09 *p* = 0.9, sex x TRF x time x genotype *χ2*_(2,135)_ = 0.6 *p* = 0.4, sex x time x genotype x diet *χ2*_(2,135)_ = 6.7 *p* = 0.03, sex x TRF x time x genotype *χ2*_(2,135)_ = 0.1 *p* = 0.9, TRF x diet x time x genotype *χ2*_(2,135)_ = 0.9 *p* = 0.6, sex x TRF x diet x time x genotype *χ2*_(2,135)_ = 0.3 *p* = 0.8. There is no effect of single factors except time. There is an interaction of sex with time, with time and genotype, diet and time, and with diet+time+genotype. The effect of genotype is only significant when interacting with the sex factor. Pairwise comparisons with post-hoc Tukey’s test indicated no significant effect of sex between groups at each time points. HF: high fat/sugar diet, NC: normal chow. Ad lib: ad libitum, TRF: time restricted feeding.

b. Distance travelled in the open field in males and females as a function of diet change (between week 1 and 8) and regimen change (between week 8 and 12). Data are means ± SEM. Repartition of 45 WT (GR^S134/S134^) homozygotes is as follows: n = 6 males + 6 females with NC ad libitum, 7 males + 7 females with HF ad libitum, 6 males + 6 females with NC restricted, 7 males + 4 females with HF restricted. Repartition of 43 mutants (GR^A134/A134^) homozygotes is as follows: n = 5 males +5 females 5 KI males with NC ad libitum, 4 males + 6 females with HF ad libitum, 5 males + 4 females with NC restricted, 7 males + 6 females with HF restricted. Data has normal distribution (Shapiro-Wilk test *p* = 0.07). The use of the chi-square distribution instead of the F-distribution is justified by the lack of homoscedasticity (Levene’s test *p* = 0.02). Five-way ANOVA (model type III): Chisq analysis: Effect of sex *χ2*_(1,78)_ = 0.01 *p* = 0.8, diet *χ2*_(1,93)_ = 2.1 *p* = 0.1, TRF *χ2*_(1,93)_ = 0.4 *p* = 0.5, time *χ2*_(1,93)_ = 2.4 *p* = 0.29, genotype *χ2*_(1,93)_ = 1 *p* = 0.3, diet x sex *χ2*_(1,93)_ = 0.5 *p* = 0.4, sex x TRF *χ2*_(1,93)_ = 0.01 *p* = 0.8, diet x TRF *χ2*_(1,93)_ = 0.2 *p* = 0.6, sex x time *χ2*_(2,129)_ = 0.2 *p* = 0.8, diet x time *χ2*_(2,129)_ = 1.8 *p* = 0.3, TRF x time *χ2*_(2,129)_ = 1.5 *p* = 0.9, sex x genotype *χ2*_(1,93)_ = 0.28 *p* = 0.59, diet x genotype *χ2*_(1,93)_ = 2.5 *p* = 0.1, TRF x genotype *χ2*_(1,93)_ = 0.09 *p* = 0.7, genotype x time *χ2*_(2,129)_ = 1.2 *p* = 0.5, sex x diet x TRF *χ2*_(1,93)_ = 0.1 *p* = 0.6, sex x diet x time *χ2*_(2,129)_ = 0.8 *p* = 0.6, sex x TRF x time *χ2*_(2,129)_ = 0.4 *p* = 0.8, diet x TRF x time *χ2*_(2,129)_ = 0.9 *p* = 0.6, sex x diet x genotype *χ2*_(1,93)_ = 0.9 *p* = 0.3, sex x TRF x genotype *χ2*_(1,93)_ = 0.001 *p* = 0.9, diet x genotype x TRF *χ2*_(1,93)_ = 0.4 *p* = 0.5, sex x genotype x time *χ2*_(2,129)_ = 1.2 *p* = 0.5, genotype x diet x time *χ2*_(2,129)_ = 1.8 *p* = 0.4, genotype x TRF x time *χ2*_(2,129)_ = 0.5 *p* = 0.7, sex x diet x TRF x time *χ2*_(2,129)_ = 0.1 *p* = 0.9, sex x diet x TRF x genotype *χ2*_(1,93)_ = 0.6 *p* = 0.6, sex x time x genotype x diet *χ2*_(2,129)_ = 2.4 *p* = 0.3, sex x TRF x time x genotype *χ2*_(2,129)_ = 0.2 *p* = 0.9, TRF x diet x time x genotype *χ2*_(2,129)_ = 0.6 *p* = 0.7, sex x TRF x diet x time x genotype *χ2*_(2,129)_ = 0.04 *p* = 0.9. There is no effect of single factors nor interactions between factors. HF: high fat/sugar diet, NC: normal chow. Ad lib: ad libitum, TRF: time restricted feeding.

**c.** % time spent in the center of the open field in males and females as a function of diet change (between week 1 and 8) and regimen change (between week 8 and 12). Data are means ± SEM. Repartition of 45 WT (GR^S134/S134^) homozygotes is as follows: n = 6 males + 6 females with NC ad libitum, 7 males + 7 females with HF ad libitum, 6 males + 6 females with NC restricted, 7 males + 4 females with HF restricted. Repartition of 43 mutants (GR^A134/A134^) homozygotes is as follows: n = 5 males + 5 females 5 KI males with NC ad libitum, 4 males + 6 females with HF ad libitum, 5 males + 4 females with NC restricted, 7 males + 6 females with HF restricted. Data has homogeneity of variance (Levene’s test *p* = 0.24). The use of the chi-square distribution instead of the F-distribution is justified by the lack of normality (Shapiro-Wilk test *p* < 0.0001). Five-way ANOVA (model type III): Chisq analysis: Effect of sex *χ2*_(1,78)_ = 0.7 *p* = 0.3, diet *χ2*_(1,78)_ = 0.6 *p* = 0.4, TRF *χ2*_(1,78)_ = 0.1 *p* = 0.7, time *χ2*_(1,78)_ = 1.1 *p* = 0.5, genotype *χ2*_(1,78)_ = 0.4 *p* = 0.4, diet x sex *χ2*_(1,78)_ = 0.05 *p* = 0.8, sex x TRF *χ2*_(1,78)_ = 0.2 *p* = 0.6, diet x TRF *χ2*_(1,78)_ = 0.2 *p* = 0.6, sex x time *χ2*_(2,110)_ = 2 *p* = 0.3, diet x time *χ2*_(2,110)_ = 1 *p* = 0.5, TRF x time *χ2*_(2,110)_ = 0.02 *p* = 0.9, sex x genotype *χ2*_(1,110)_ = 0.9 *p* = 0.3, diet x genotype *χ2*_(1,78)_ = 0.15 *p* = 0.6, TRF x genotype *χ2*_(1,78)_ = 0.04 *p* = 0.8, genotype x time *χ2*_(2,110)_ = 0.4 *p* = 0.8, sex x diet x TRF *χ2*_(1,78)_ = 0.05 *p* = 0.8, sex x diet x time *χ2*_(2,110)_ = 1 *p* = 0.6, sex x TRF x time *χ2*_(2,110)_ = 0.4 *p* = 0.7, diet x TRF x time *χ2*_(2,110)_ = 0.3 *p* = 0.8, sex x diet x genotype *χ2*_(1,78)_ = 0.08 *p* = 0.7, sex x TRF x genotype *χ2*_(1,78)_ = 0.07 *p* = 0.7, diet x genotype x TRF *χ2*_(1,78)_ = 0.09 *p* = 0.7, sex x genotype x time *χ2*_(2,110)_ = 2.5 *p* = 0.2, genotype x diet x time *χ2*_(2,110)_ = 0.01 *p* = 0.9, genotype x TRF x time *χ2*_(2,110)_ = 0.06 *p* = 0.9, sex x diet x TRF x time *χ2*_(2,110)_ = 0.3 *p* = 0.8, sex x diet x TRF x genotype *χ2*_(1,78)_ = 0.09 *p* = 0.7, sex x time x genotype x diet *χ2*_(2,110)_ = 0.6 *p* = 0.7, sex x TRF x time x genotype *χ2*_(2,110)_ = 0.8 *p* = 0.6, TRF x diet x time x genotype *χ2*_(2,110)_ = 0.1 *p* = 0.9, sex x TRF x diet x time x genotype *χ2*_(2,110)_ = 0.3 *p* = 0.8. There is no effect of single factors nor interactions between factors. HF: high fat/sugar diet, NC: normal chow. Ad lib: ad libitum, TRF: time restricted feeding.
